# Unveiling the Collaborative Effect at the Cucurbit[8]uril‐MoS_2_ Hybrid Interface for Electrochemical Melatonin Determination

**DOI:** 10.1002/chem.202203244

**Published:** 2023-01-13

**Authors:** Rut Martínez‐Moro, María del Pozo, Jesús I. Mendieta‐Moreno, Alba Collado, Sofia Canola, Luis Vázquez, María Dolores Petit‐Domínguez, Elena Casero, Carmen Quintana, José A. Martín‐Gago

**Affiliations:** ^1^ Departamento de Química Analítica y Análisis Instrumental Facultad de Ciencias Universidad Autónoma de Madrid c/Francisco Tomás y Valiente, N° 7 Campus de Excelencia de la Universidad Autónoma de Madrid 28049 Madrid Spain; ^2^ Institute of Physics of the Czech Academy of Sciences Cukrovarnicka 10 Prague 6 CZ 162 00 Czech Republic; ^3^ Departamento de Física Teórica de la Materia Condensada Facultad de Ciencias Universidad Autónoma de Madrid c/Francisco Tomás y Valiente, N° 7 Campus de Excelencia de la Universidad Autónoma de Madrid 28049 Madrid Spain; ^4^ Departamento de Química Inorgánica Facultad de Ciencias Universidad Autónoma de Madrid c/Francisco Tomás y Valiente, N° 7 Campus de Excelencia de la Universidad Autónoma de Madrid 28049 Madrid Spain; ^5^ ESISNA group Instituto de Ciencia de Materiales de Madrid (CSIC) c/Sor Juana Inés de la Cruz 3 Campus de Excelencia de la Universidad Autónoma de Madrid 28049 Madrid Spain

**Keywords:** 2D-MoS_2_, cucurbit[8]uril, melatonin, density functional theory (DFT), supramolecular chemistry

## Abstract

Host‐guest interactions are of paramount importance in supramolecular chemistry and in a wide range of applications. Particularly well known is the ability of cucurbit[*n*]urils (CB[*n*]) to selectively host small molecules. We show that the charge transfer and complexation capabilities of CB[*n*] are retained on the surface of 2D transition metal dichalcogenides (TMDs), allowing the development of efficient electrochemical sensing platforms. We unveil the mechanisms of host‐guest recognition between the MoS_2_‐CB[8] hybrid interface and melatonin (MLT), an important molecular regulator of vital constants in vertebrates. We find that CB[8] on MoS_2_ organizes the receptor portals perpendicularly to the surface, facilitating MLT complexation. This advantageous adsorption geometry is specific to TMDs and favours MLT electro‐oxidation, as opposed to other 2D platforms like graphene, where one receptor portal is closed. This study rationalises the cooperative interaction in 2D hybrid systems to improve the efficiency and selectivity of electrochemical sensing platforms.

## Graphical abstract

**The adsorption geometry of cucurbit[8]uril** on a 2D nanoplatform controls the recognition capabilities. It behaves like a traffic light: on a transition metal dichalcogenide, the reception portals of CB[8] are open and the guest molecule can fit in. On graphene (or carbonaceous termination) they are closed, which prevents complex formation.

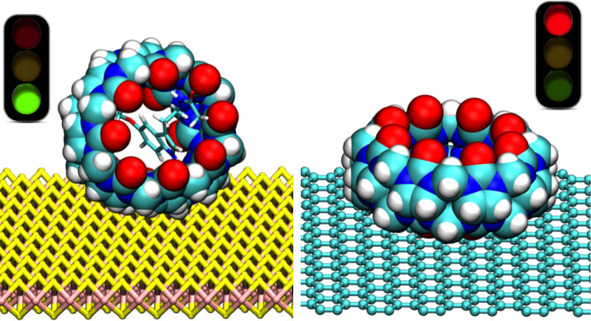

## Introduction

Selective encapsulation of small guest molecules enables the development of controlled supramolecular architectures with interesting properties in a wide range of applications. Cucurbit[*n*]urils (CB[*n*]s)_*n*=5–10_ constitute an important macrocyclic family with improved selectivity towards analytes through host‐guest interactions driven by its particular molecular structure.[^1^, ^2^] Their nonpolar cavity allows the formation of inclusion complexes with hydrophobic or relatively hydrophobic guests of an appropriate size, whereas the presence of carbonyl groups at the portals allows electrostatic or ion‐dipole interactions with positively charged analytes, thus leading to the formation of different adducts.[^3^, ^4^, ^5^, ^6^, ^7^] On the other hand, transition metal dichalcogenides (TMDs) nanosheets are 2D graphene‐like materials proposed for many applications. In this article, we reveal that the selective host‐guest recognition capabilities of CB[*n*] are retained at the hybrid interface with 2D TMDs. The charge transfer to/from the hosted molecule is efficient and the selectivity of the guest‐host system is retained, and therefore, this platform can be used as an improved electrochemical sensor. Moreover, it has been recently demonstrated synergetic effects when TMDs are combined with other receptors in electrochemical sensors, amplifying the voltametric response of the system.[^8^, ^9^, ^10^, ^11^] In this work we unveil the fundamental mechanisms responsible for this effect.

To this aim, we use the hybrid interface formed by Cucurbit[8]uril (CB[8]) and MoS_2_ to selectively amplify the signal produced by hosted *N*‐acetyl‐5‐methoxytryptamine, best known as melatonin (MLT), an endogenous hormone derived from serotonin synthesized in the pineal gland in vertebrates. MLT is of paramount significance in organisms since it regulates circadian rhythms and blood pressure, acts as antioxidant and anti‐inflammatory agent, and is a free radical scavenger playing therefore, an important role in cell protection.[^12^, ^13^, ^14^] Such a wide scope has made that recent applications are focused on MLT analysis in food,[^15^, ^16^, ^17^] pharmaceutical[^16^, ^17^, ^18^] or biological samples as urine, saliva and nails.[^19^, ^20^, ^21^, ^22^] Therefore, various analytical methods have been developed for MLT quantification. Although different chromatographic techniques with UV‐vis,^[18]^ fluorescence^[15]^ or mass spectrometry detection^[21]^ have been proposed, electrochemical methods result an interesting alternative to these expensive and sophisticated techniques. In contrast, the electrochemical ones offer a high sensitivity with cheaper and simpler instrumentation. In the literature, it is possible to find strategies using a bare glassy carbon electrode,^[17]^ or using novel nanomaterials as modifiers of the electrode surface,[^16^, ^19^, ^23^] resulting an excellent alternative to increase the sensitivity and selectivity of the determination.

In this work, we combine experimental techniques, such as nuclear magnetic resonance spectroscopy (NMR) and atomic force microscopy (AFM), and theoretical methods based on the density functional theory (DFT) combined with molecular dynamics (MD) calculations, to unveil the adsorption molecular structure on the hybrid interface that leads to an enhancement of the electrochemical signal of the MLT oxidation. The good match between theory and experiments is due to the fact that our calculations are performed in liquid environment, which underline the important role played by the solvation and the need of including water molecules in the calculations for reproducing the experiments and understanding the chemical mechanisms. We conclude that CB[8] adsorbs robustly on the TMDs surface letting the cavity open for complex formation with MLT. This situation is reversed in other carbonaceous surfaces, where the CB[8] receptor portals are closed hindering the complex formation. This study may foster the use of TMDs in supramolecular recognition nanoarchitectures and it paves the way to the use of hybrid CB[n]‐low dimensional systems as excellent platforms to develop, for instance, electrochemical sensors applied in real analysis.

## Results and Discussion

### Structural Interaction between MLT and CB[8]

**Figure 1:**
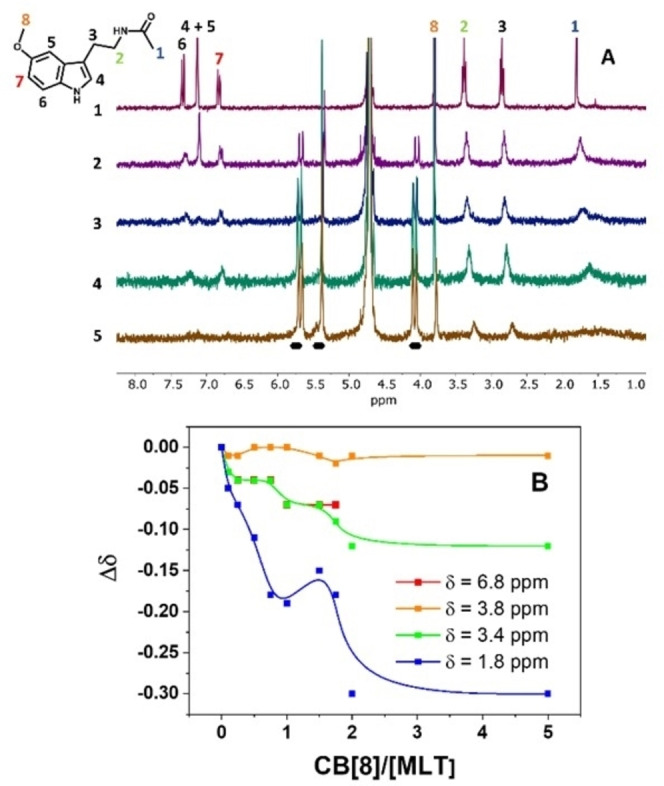
NMR results of the complex CB[8] with MLT. A) ^1^H NMR spectra obtained from titration experiments of 2.0 mM MLT (spectrum 1, signal assignment in MLT structure) with CB[8] (0.10–5.0 equiv, from 2 to 5) in D_2_O. The CB[8] proton signals are indicated in the figure by black marks. B) Fitting of selected protons shift change with CB[8] concentration.

CB[8]/MLT formation was studied by ^1^H NMR spectroscopy titration analysis. This technique allows to determine the interaction of MLT and CB[8] since a MLT proton signal upfield shift is expected when the inclusion complex formation occurs due to the interaction with the CB[8] internal cavity.^[24]^ With this aim, increasing amounts of CB[8] (0.10–5.0 equivalents relative to MLT) were added to a constant concentration of MLT in D_2_O and the ^1^H NMR spectra were recorded. As shown in Figure 1, upon addition of CB[8], broad resonances become predominant in the spectra which indicates that complexation occurs under a fast exchange relative to the NMR timescale. Moreover, most of the MLT signals were slightly shifted upfield due to the interaction of MLT with the CB[8] cavity.

The MLT aromatic proton signals appearing at 7.3, 7.2, and 6.8 ppm, (peaks labeled from 4 to 7 in Figure 1A) and the methylene proton signals at 2.8 and 3.4 ppm (labeled as 2 and 3) undergo a very similar shift while the signal at 1.8 ppm (peak 1), corresponding to the acetamide CH_3_ group, shows the largest shift, suggesting a stronger interaction of this group with the CB[8] unit. Additionally, the signal corresponding to the MLT methoxy group (3.8 ppm, peak 8) is slightly affected upon CB[8] addition, in terms of both signal shift and broadness, which suggests that this methoxy group is placed outside of the cavity and does not interact with the carbonyl groups of the CB[8] portal.

The variation of the NMR signal upon formation of the complex CB[8]/MLT can be understood from MD with quantum mechanics/molecular mechanics (QM/MM) approach, in which the water environment has been included.^[25]^

Figure1B shows the correlation between the signal shift (▵δ) for selected MLT protons and the CB[8] ratio extracted from the NMR spectra. For clarity, only the most relevant signals are shown. The signal does not change linearly suggesting the formation of two different stoichiometries of the CB[8]/MLT complex (See Figure S1 and discussion on one vs. two molecules inside the cage in the Supporting Information).

**Figure 2:**
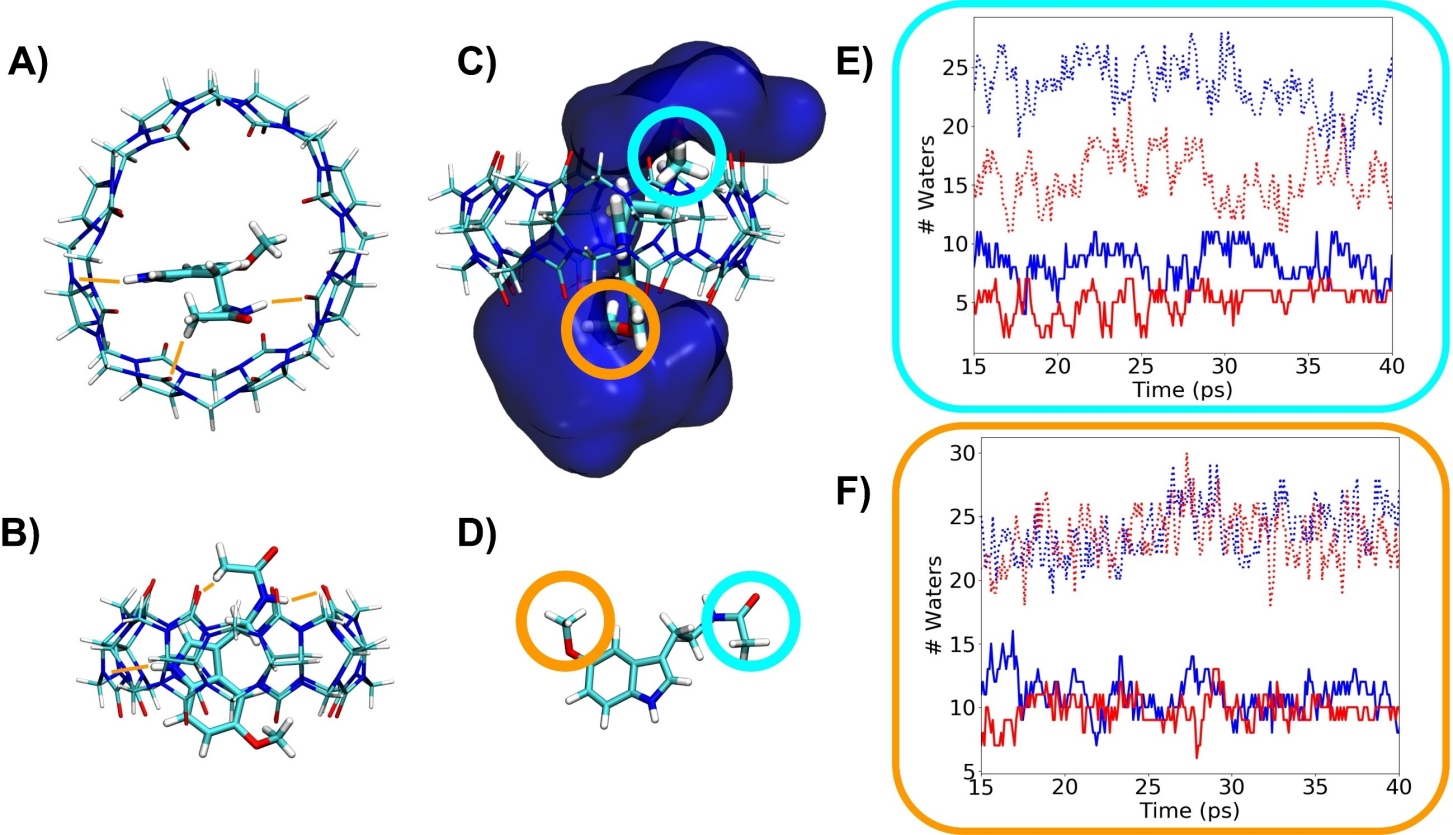
Conformation structure of the complex CB[8]/MLT surrounded by water. A), B) Structure of the complex CB[8]/MLT, represented without the water molecules for clarity, from two different perspectives. The stabilizing interactions are represented in orange. C) Structure of the complex CB[8]/MLT including water molecules. The dark blue surface represents the density of water molecules at a distance of 5 Å from MLT, which creates a channel inside the CB[8] cage. D) Schematic representation of the melatonin marking the two aliphatic groups. An orange line encircles the methoxy and light‐blue the acetamide groups. E) Evolution of the number of water molecules around the acetamide CH_3_ group in the first (solid lines) and second (dashed lines) water shells for the complex CB[8]/MLT (red) or noncomplexed MLT (blue). F) Evolution of the number of waters around the methoxy group in first (solid lines) and second (dashed lines) water shell for the complex CB[8]/MLT (red) or noncomplexed MLT (blue).

We determined that the complex CB[8] with one single MLT is the most stable configuration. The formation energy gain is about 0.99 eV with respect to two molecules inside the CB[8] cage (see the Supporting Information). This low‐energy configuration, together with the solvation environment, is shown in Figure 2A–C, where we have removed the water molecules for a better visualization of the structure in panels A and B. Figure 2C shows the position of the water molecules inside the cage as a blue channel through the center of the complex that corresponds to a column of water inside the cucurbituril. The presence of this water channel gathered in the middle of the cavity accounts for the hydrophobic nature of the CB[8]. This figure shows the different interaction of water at both terminations of MLT, responsible for the ▵*δ* of Figure1B. To be more quantitative in this analysis, for both MLT and the complex CB[8]/MLT, we have determined the first and second water shells for both methyl groups, acetamide (blue circle in Figure 2C and 2D and labeled as 1 in Figure 1) and methoxy (orange circle in Figure 2C and 2D and labeled as 8 in Figure 1). In these analyses, we observe that for the CH_3_ in acetamide group (Figure 2E) there is a difference in the average non‐complexed MLT (blue line) or in the complex (red line). In this case, the average number of water molecules for the first shell is 5.1 in complex and 8.3 in water. Same behavior is observed with the second water shell (dashed lines) with an average of water molecules of 15.7 in complex and 23.6 in water. However, if we analyze the methoxy group (Figure 2F), we observe that it is much less affected in the complex. The first shell has an average number of water molecules of 9.8 in complex and 10.6 in water, and the second 24.0 and 23.6, respectively. This change in behavior of both aliphatic groups can be rationalized with the interaction of acetamide NH of the MLT group with CB[8] that locally changes the water environment (see orange lines in Figure 2A–B), for the stability of these interactions see Figure S2. The methoxy group instead is interacting with the water channel formed inside the complex (Figure 2C) and does not experience any chemical shift. Summarizing, we can depict from our calculations that the acetamide group (labeled as 1 in Figure1 A) shall experiment a clear shift in the NMR, whereas the methoxy group (labeled as 8 in Figure1 A) shall not change its resonance frequency, in good agreement with Figure 1B.

### CB[8]‐MoS_2_ surface interaction

**Figure 3:**
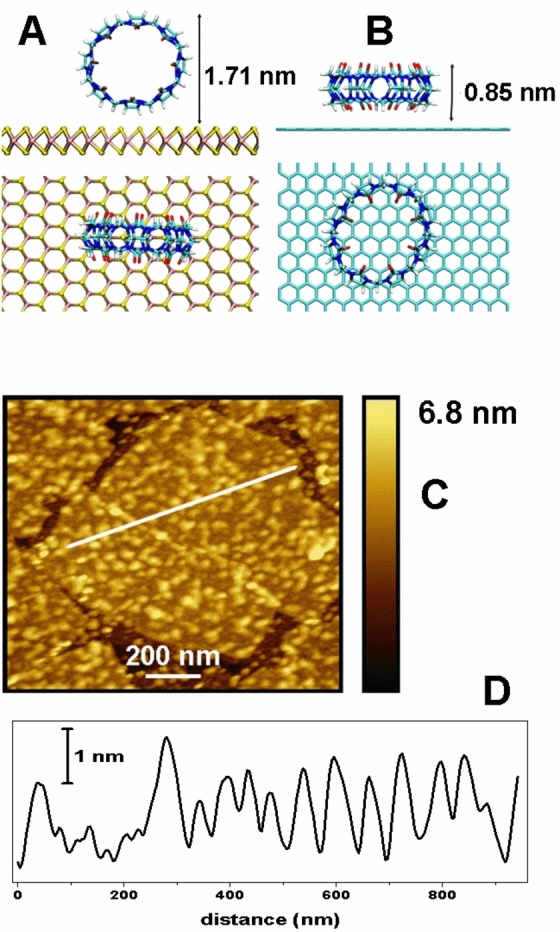
Structure of adsorption of CB[8] on different surfaces. DFT‐calculated adsorption geometries of CB[8] on the A) MoS_2_ and B) graphene surfaces. C) AFM image of a MoS_2_ flake on Si covered by CB[8]. D) Surface profile along the line drawn in (C).

The remarkable chemical affinity of CB[*n*]s makes them excellent compounds to increase the selectivity of electrochemical sensors. To this end, we have designed a system consisting of CB[8] adsorbed on the surface of a TMD (MoS_2_) nanosheet, which was previously deposited on the GC electrode surface. The election of a TMD 2D platform is not arbitrary. First, it has shown excellent synergetic results towards determination of other analytes[^10^, ^11^, ^26^] and second, the chalcogenide termination induces a selectivity in the surface molecular adsorption. We have performed DFT geometrical relaxations and calculated the minimum energy for different geometries showing that the adsorption of a CB[8] on a TMD takes place with the plane defined by the oxygens perpendicular to the surface (both receptor portals are open), as indicated in Figure 3A. This adsorption geometry is more stable than the one obtained with the CB[8] oxygens bounded to the TMD surface (one portal is closed), by an energy difference of 0.94 eV. In this particular configuration there is space for the MLT to accommodate inside the CB[8]. The structure is stabilized by the interaction between the H atoms of the CB[8] and the S atoms of the MoS_2_ surface, with a distance of 2.4 Å between the closest H and the S atoms. Note that the CB[8] is adsorbed in a quasimolecular form as the cage has been only slightly deformed by the adsorption process (the diameter of the cage in the direction perpendicular to the surface is about a 15 % smaller than along the surface) and the structural values are in agreement with previous publications.^[27]^

AFM analysis confirms this theoretical finding. Figure 3C shows an AFM image of CB[8] adsorbed on MoS_2_ flakes on a Si wafer. This sample has been prepared following the same modification procedure as for the electrochemical sensor preparation. Si surfaces have been used instead of GC to reduce the surface roughness, which will help to a better identification of the molecules. The surface profile shown in Figure 3D shows that the MoS_2_ flakes are covered by small independent nanostructures with typical height in the 1–3 nm range. They cover around the 66 % of the MoS_2_ flakes. The height of these structures is compatible with the calculated vertical adsorption geometry depicted in Figure 3A and therefore, they correspond to a molecular layer or individual CB[8] molecules.

We analysed the electrostatic potential of CB[8] in MoS_2_ surface (Figure S3), and we have not found any remarkable polarization in CB[8]. We suggest that the main role of the surface is enhancing the signal.

**Figure 4:**
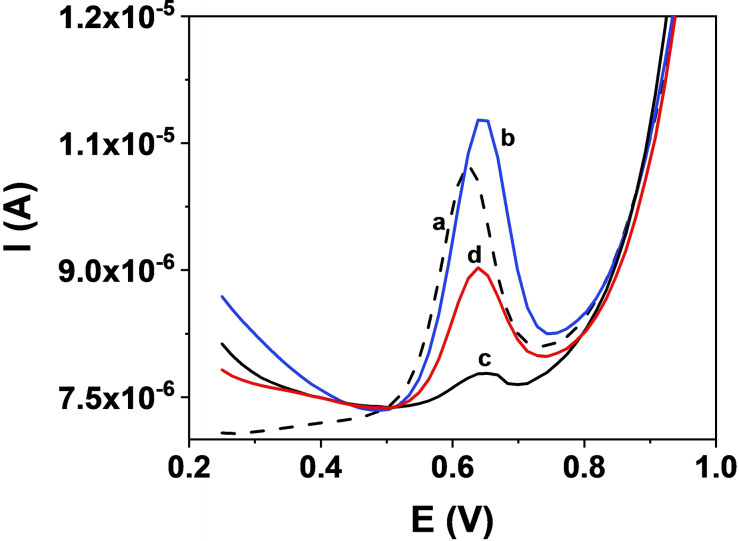
Differential pulse voltametric response to different electrodes. a) bare GC, b) GC/MoS_2_/CB[8], c) GC/Gox/CB[8] and d) GC/Gox_1:10 diluted_/CB[8] systems

Furthermore, we have calculated the adsorption geometry of the CB[8] on other carbon‐based surfaces, as graphene (Figure 3B). Interestingly, we observe that on carbon, the interaction consists of dispersive forces and, therefore, the horizontal geometry where the oxygens point to carbon atoms (one portal closed) is preferred to the one with both portals open by 2.13 eV. This geometry hampers the inclusion of the MLT in the complex (Figure 4c and d). We have also determined the adsorption geometry in graphite by AFM, where CB[8] species aggregate in islands with a typical height of 0.6 nm, compatible with the molecular adsorption structure depicted by calculations (see Figure 3B and the Supporting Information).

### Synergetic effect in the electrochemical sensor

The different CB[8] adsorption geometry for TMDs and carbonaceous surface is corroborated by recording the electrochemical response of the MLT oxidation using electrochemical sensors prepared with the hybrid 2D/CB[8] materials discussed above. Figure 4 shows the differential pulse voltammetry (DPV) curves. The MLT oxidation response obtained with the bare GC electrode (voltammogram a) is also included as reference.

In this later case, a wave at around 0.60 V is clearly observed as previously described for this system.^[16]^ What is noticed, is the difference in the responses of MLT with the sensors resulting from the modification with the corresponding 2D material (TMD or graphene oxide, Gox) and CB[8]. While a clear increase in the current is produced with the GC/MoS_2_/CB[8] system (voltammogram b), just the opposite effect is observed when the TMD is substituted by Gox (voltammograms c and d where different amounts of Gox are employed). The results obtained with Gox, a peak potential shift to higher values and a decrease in the current, are those usually observed when macrocyclic receptors are employed for electrochemical sensors development.[^28^, ^29^] As depicted in our model, the synergetic effect produced by the MoS_2_/CB[8] combination leading to higher oxidation current, is driven by the adsorption geometry of the CB[8] on the TMD surface, which leaves open the portals for entrance of the guest molecule. This geometry is very specific of the combination of CB[n] with TMDs and does not work on other surfaces where the interaction may be driven by dispersion forces between them, as carbon terminated surfaces.

The electrochemical oxidation mechanism of MLT on a GC electrode has been described in the scientific literature.[^17^, ^30^, ^31^, ^32^] The oxidation process in a GC/MoS_2_/CB[8] is observed in the voltammogram at around 0.66 V, that is a higher voltage than that required for the oxidation of the “noncomplexed” MLT in a GC/MoS_2_ electrode (Figure S4). These voltage values are related to the energy required to remove electrons from MLT.^[33]^ Accordingly, calculations (see Table SI_T1 and discussion in 1.2 of the Supporting Information) show that the complex formation originates a small shift towards higher values of the oxidation energies for MLT, in line with the experimental observation in the voltammograms in Figures 4 and S4. The electrochemical signal of the MLT oxidation involves the amino group of the structure.^[17]^ In Figure 2A it is visible that the acetamide group is establishing a H‐bond with the oxygens of the CB[8]. This interaction stabilizes the complex and, at the same time, is approaching the MLT to the CB[8]/TMD surface making easier the charge transfer (Figure 4b).

**Figure 5:**
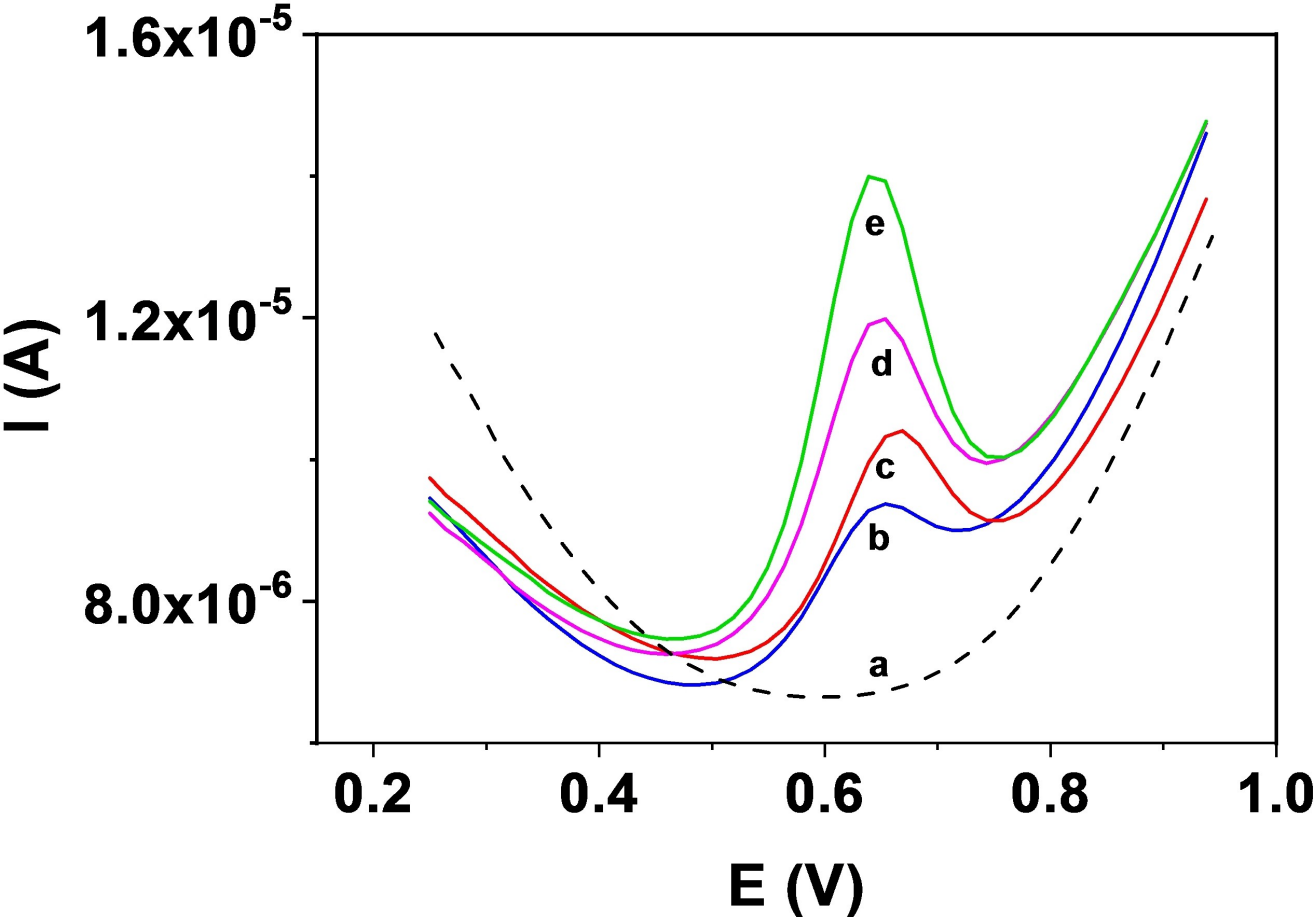
Voltammetric response of GC/MoS_2_/CB[8] with increasing [MLT]. a) 0.2 M pH 7.0 phosphate buffer b) [MLT]=5×10^−6^ M; c) [MLT]=1×10^−5^ M; d) [MLT]=2×10^−5^ M; e) [MLT]=3.5×10^−5^ M.

Once proved the synergetic effect of the combination MoS_2_/CB[8], we tested the viability of using this system in an electrochemical sensor for the electroanalytical quantification of MLT. As depicted in Figure 5, this sensing platform responds linearly to MLT concentration increasing the oxidation current recorded for increasing MLT amounts.

## Conclusions

By using molecular dynamics with quantum mechanics/molecular mechanics and including surrounding water molecules, we have rationalized that one melatonin inside the CB[8] is stabilized by the formation of a water channel. This MLT interacts with the CB[8] via the amide group through a H‐bond, being accessible for the oxidation detected in the voltammograms. The enhanced specificity of the complex makes this system an advanced platform for electrochemical determination of this important molecule. Our calculations, performed in a liquid environment, perfectly match NMR data, and underline the important role played by the solvation and the need to include water molecules in calculations for reproducing experiments and understanding chemical mechanisms. It is important to note that often accurate calculations are performed in a vacuum, and they underestimate the effect of the liquid environment.

These results can be directly generalized to other 2D TMDs and analytes and suggest that previously reported DPV signal amplification is a synergetic effect related to a particular adsorption structure of the receptors in electrodes. Electrochemical techniques have scarcely been explored for MLT analysis despite their several advantages such as fast data acquisition or cheap and easy handling equipment. They are usually based on the detection of MLT oxidation on different carbon electrode surfaces, sometimes modified with nanomaterials, and open new and exciting possibilities in the development of innovative electrochemical sensors that combine nanomaterials and macrocyclic molecular receptors.

## Author Contributions

R.M.‐M. and M.P. made the electrochemical experiments, L.V. recorded and analysed AFM images, S.C., and J.M. performed the calculations, A.C. performed the NMR experiments. All authors discussed the results and participated in the paper preparation and editing. The research was coordinated by J.A.M.‐G., E.C., M.D.P.‐D. and C.Q.

## Conflict of interest

The authors declare no conflict of interest.

1

## Supporting information

As a service to our authors and readers, this journal provides supporting information supplied by the authors. Such materials are peer reviewed and may be re‐organized for online delivery, but are not copy‐edited or typeset. Technical support issues arising from supporting information (other than missing files) should be addressed to the authors.

Supporting Information

## Data Availability

The data that support the findings of this study are available from the corresponding author upon reasonable request.
