# Supplementary material for: Unveiling the Collaborative Effect at the Cucurbit[8]uril‐MoS2 Hybrid Interface for Electrochemical Melatonin Determination
Source: Chemistry. 2023 Jan 13;29(9):e202203244. doi: 10.1002/chem.202203244 (PMC10107440; doi:10.1002/chem.202203244)
Supplement: Supplementary file 1 — Supporting Information [file CHEM-29-0-s001.pdf]

Chemistry–A European Journal

Supporting Information

Unveiling the Collaborative Effect at the Cucurbit[8]uril-MoS_2 Hybrid Interface for Electrochemical Melatonin Determination

Rut Martínez-Moro, María del Pozo, Jesús I. Mendieta-Moreno, Alba Collado, Sofia Canola, Luis Vázquez, María Dolores Petit-Domínguez, Elena Casero, Carmen Quintana,* and José A. Martín-Gago*

Table of Contents

1. Materials and Methods……………………………………………………………………………………………………3

2. Procedures……3

3. Further DFT and QM/MM MD calculations……………………………………………………………………………3

3.1. Can be two MLT molecules inside the CB[8] cage?..3

3.2. Cucurbituril MoS_2 interface …………………..……..4

3.3. Estimation of the oxidation energy variation...5

4. CB[8] on graphite by AFM……………………………………………………………………………………………..6

1. Materials and Methods

Reagents and Instrumentation. Sigma Aldrich supplied molybdenum disulphide (99%, 90 nm powder), Cucurbit[8]uril, and Melatonin (MLT) (99%). Deuterium oxide, ethanol absolute (EtOH) (99%), and phosphoric acid were purchased from Scharlau (Barcelona, Spain). All reagents used were of analytical reagent grade. Milli-Q water was purified with a Milli Ro Milli Q Plus 185 apparatus from Millipore (Waters, Milford, USA).
Electrochemical measurements were performed with a μ-Autolab Type III potentiostat employing Gpes software (both from Metrohm Autolab, Utrecht, Netherlands). A conventional three-electrode system was employed, with a glassy carbon electrode (GC; 3 mm in surface diameter) as the working electrode (bare or modified either with MoS_2 nanosheets (GC/MoS_2) or with both materials (GC/ MoS_2/CB[8]), an Ag/AgCl/KCl (3M) as the reference electrode, and a coiled platinum wire as the counter one.
1H-NMR spectra (2.0 mM MLT and increasing concentrations of CB[8]) were recorded using a Bruker Avance-300 spectrometer (300 MHz). The solutions of MLT and MLT with increasing concentration of CB[8] were prepared in D_2O. The solution of CB[8] was subjected to ultrasound.
Atomic Force Microscopy characterization was done with a Nanoscope IIIa system (Veeco). The images were analyzed with the Gwyddion free-software package. [1]

Calculations. The internal energy and geometry of the MLT and CB[8] complexes have been calculated with a QM/MM approach. In the QM region we used the Fireball DFT[2] method with BLYP exchange-correlation functional[3] with D3 corrections[4] and norm-conserving pseudopotentials. We employed a basis set of optimized numerical atomic-like orbitals (NAOs) [5] with a 1s orbital for H and sp^3 orbitals for C, N and O atoms. The water molecules were included in the MM region, and they were modelled with the TIP3P approach. [6] This methodology has been used previously with successful results. [7,8] The interaction energy was calculated using MMPBSA. [9] Geometric optimizations of CB[8] in graphene and MoS_2 surfaces have been calculated with Fireball DFT. The calculation of oxidation energy variation of MLT have been performed with Gaussian16 software at the B3LYP/6-31G* level of theory [10], with D3BJ empirical dispersion[4] and inclusion of the water solvent effect through the polarizable continuum model. [11] The effect of complexation on MLT electronic properties is included by modeling CB[8] with partial electrostatic charges provided from a Fireball calculation of CB[8] on MoS_2.

2. Procedures

Nanomaterials synthesis. MoS_2 nanosheets were prepared by liquid exfoliation following a procedure previously reported. [12] Briefly described: 75 mg of MoS_2 are exfoliated in 10 mL of EtOH/H_2O (45/55, v/v) by ultrasonication during 2 h. The suspension was kept 24 h at 4ºC and next centrifuged at 4000 rpm for 45 minutes. Finally, the supernatant is separated and stored at 4ºC. 0.3 mM CB[8] suspensions are prepared in ultrapure water.

Sensor preparation. The electrode surface was layer by layer modified by drop-casting procedure with first, 6 µL of the MoS_2 synthetized 2D material, followed by 6 µL of the CB[8] suspension (GC/MoS_2/CB[8] system). After each modification step, the corresponding electrode surface was let drying at 40ºC. For comparison, the same procedure was carried out only with the MoS_2 suspension (GC/ MoS_2).

Electrochemical measurements. Differential pulse voltammetry (DPV) scans were carried out from 0.25 to 0.95 V, in 0.2 M phosphate buffer pH 7 as supporting electrolyte at a scan rate of 30 mV/s.

Atomic Force Microscopic (AFM) measurements. The AFM measurements were done operating in the dynamic mode using silicon cantilevers with a nominal force constant in the 1-5 N/m range, and nominal radius of curvature of 8 nm. The images consisted of 512 x 512 pixels.

3. Further DFT and QM/MM MD calculations

3.1. Can be two MLT molecules inside the CB[8] cage?

To understand the interaction of MLT and CB[8] in water, we perform QM/MM [7] molecular dynamics in water, taking into account all the possible species, namely MLT and CB[8] free in solution along with the supramolecular complex including 1 or 2 MLT in CB[8]. From this simulation, we calculated the internal energy and the solvation energy of the different systems (**Figure S1**). The internal energy of the complexes has been calculated with a QM/MM approach from the last 20 ps of a QM/MM Molecular Dynamics of 40 ps for each system, with MLT and/or CB[8] in the QM region and the water molecules in the MM region.
To determine the most stable structure geometry of both complexes, we consider different initial configurations, and we compare their formation energies. In **Figure S1** it is represented the most stable complex including two (**Fig S1a**) or one (**Fig S1b**) MLT molecules. To calculate which complex is more stable at different MLT/CB[8] amount ratios, we compare two different cases.

First, we consider the case of MLT and CB[8] in a relation 1/1 and we compare the difference in energy between the complex of 2 MLTs in one CB[8] (2MLT/CB[8]) + CB[8] alone vs twice one MLT in CB[8]):
2MLT/CB[8] +CB[8] > 2*(1MLT/CB[8])   <u>relation 1 MLT per 1 </u>CB[8]
-1265014 > -1265068 (Δ=54 kcal/mol), **Best 1 Melatonin inside the cucurbituril**

The second case is when there is an excess of melatonin, therefore we considered 2 MLTs per CB[8]. In this case the comparison is between 2 melatonin in one CB[8] (2MLT/CB[8]) vs one melatonin in CB[8] (1MLT/CB[8]) plus MLT alone):
2MLT/CB[8] < 1MLT/CB[8]+MLT <u>relation 2 melatonin per 1 </u>CB[8]
-716988 > -717019 (Δ=31 kcal/mol), **Best 1 Melatonin inside the cucurbituril**

From this calculation we can conclude that the case of one melatonin inside CB[8] (1MLT/CB[8] complex) is the most favourable in water. We observe that the complex including two MLT inside CB[8] (2MLT/CB[8]) is stable in water and even if it is not the most favourable it will be more relevant at high MLT concentration.

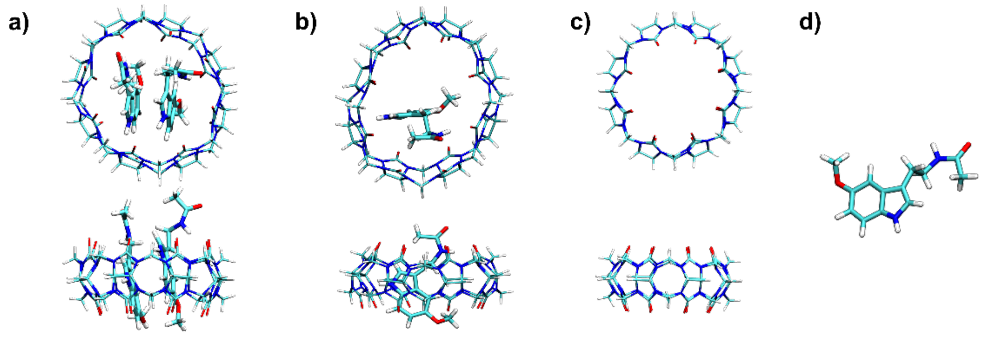

	a)	b)	c)	d)	
**Internal Energy:**	**-716512**	**-632044**	**-547532**	**-84467**	**(kcal/mol)**
**Solvation energy:**	**-476**	**-490**	**-494**	**-18**	**(kcal/mol)**
**Total:**	**-716988**	**-632534**	**-548026**	**-84485**	**(kcal/mol)**

Figure S1: Calculation of Internal and solvation energy for the complex in water with two MLT and CB[8] (2MLT/CB[8]) (a), one melatonin and CB[8] (1MLT/CB[8]) (b) CB[8] (c) and MLT (d), in water.

The interactions in orange on **Figure 2** represents the most stable interactions found during the molecular dynamic. To show the stability we plot the evolution of distance and angle during the last 20 ps of the MD in **Figure S2** where we can observe that these three interactions are stable during the last steps of the MD. We can also observe that H1 and H3 create a hydrogen bond with CB[8] and the angle is stable around 160º.

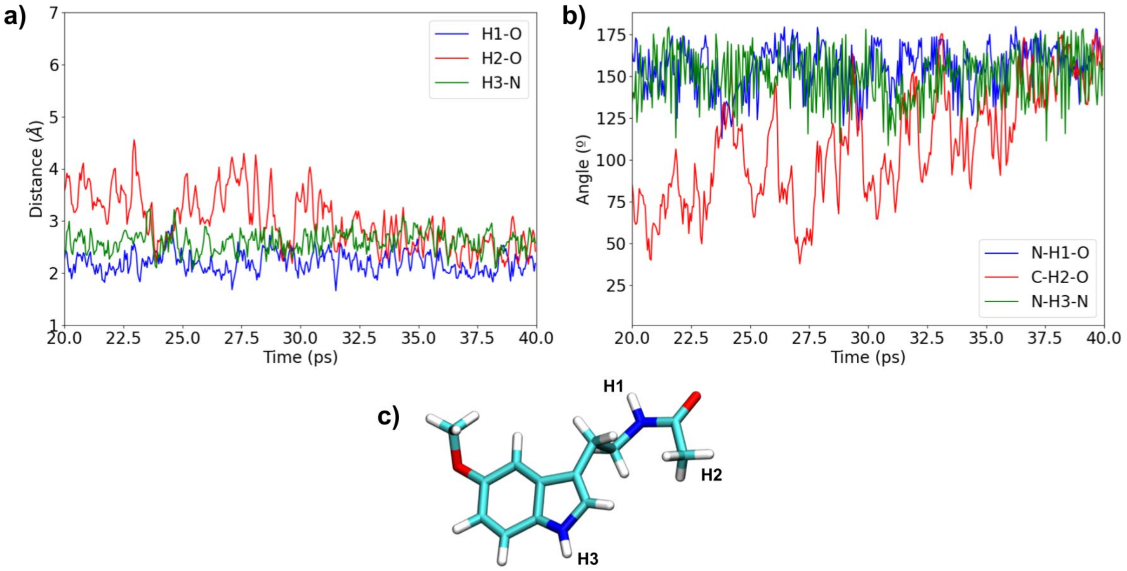

Figure S2: Distances a) and angles b) of the stabilizing interactions during the molecular dynamic of 1MLT/CB[8] complex. In the distance labels the O or N at the right corresponds to Oxigen or N of cucurbituril. In the case of the label for the evolution of the angles the atom at the left correspond to the bonded atom to the H and at the right to the atom in CB[8]. c) Scheme with the three hydrogens involved in the interactions.

3.2. Cucurbituril MoS_2 interface.

To see the effect of MoS_2 surface in CB[8] we calculated the electrostatic potential of the relaxed structure, see **Figure S3**. We can observe in Figure S3 that the polarization of CB[8] is not visible. Therefore we suggest that MoS_2 surface will not create a shift in the oxidation energy of MLT when is inside CB[8].

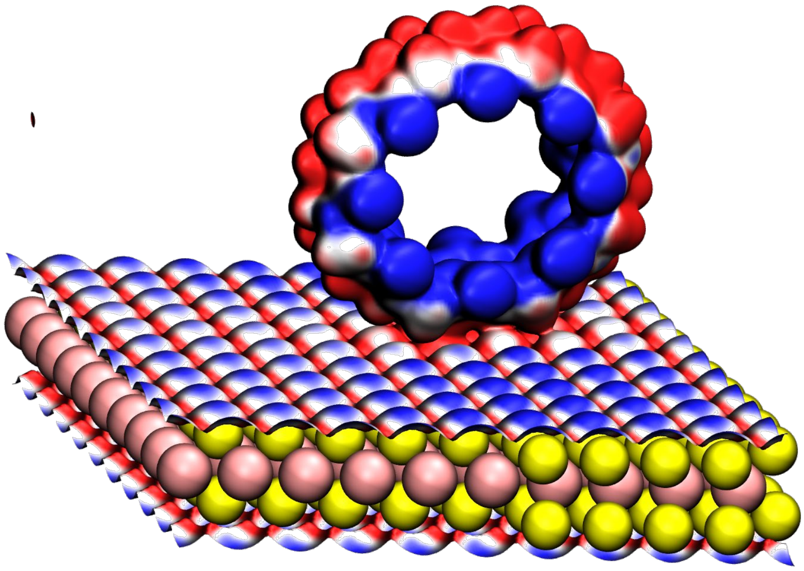

Figure S3. Electrostatic potential calculated with DFT from the low energy relaxed structure of CB[8] on MoS_2 surface.

3.3. Estimation of the oxidation energy variation

The shift of the oxidation potential upon MLT complexation with CB[8] can be due to different factors. To rationalize the change in the oxidation potential experimentally determined in **Figure S4**, we have compared the electronic structure of MLT adsorbed on MoS_2 in different geometries and environments, to account for the electrochemical experimental conditions. We performed a relaxation of MLT on MoS_2 surfaces using Fireball DFT code, [2] starting from different initial geometries. The lowest energy structure is displayed in **Figure S5** for MoS_2 (MLT/MoS_2). On the other side, MLT can be complexed inside CB[8], when CB[8] is absorbed on MoS_2 with open receptor portals (**Figure 3A**). To calculate this structure we have considered the relaxed geometry obtained with QM/MM for MLT inside

CB[8] in water (MLT/CB[8]), see **Figure S1b.** From the obtained MLT geometries MLT/MoS_2 and MLT/CB[8], a geometry optimization of neutral MLT has been performed at the B3LYP-D3BJ/6-31G* level of theory and, at the fixed neutral equilibrium geometry, the absolute energy of the neutral and cation molecule has been computed. The total energy difference between the neutral and cation molecule computed in solution can be taken as a measure of the oxidation energy of the molecule in solution, [13] which is directly related to the highest occupied molecular orbital eigenvalue (HOMO) of the molecule in solution. Our calculations (**Table S1**) allow us to conclude that the formation of the supramolecular complex slightly affects the oxidation potential, causing a small increase in the oxidation potential (shift of ca. 0.02 eV), consistent with the experimental results (shift of ca. 0.06 V, see **Figure S4**).

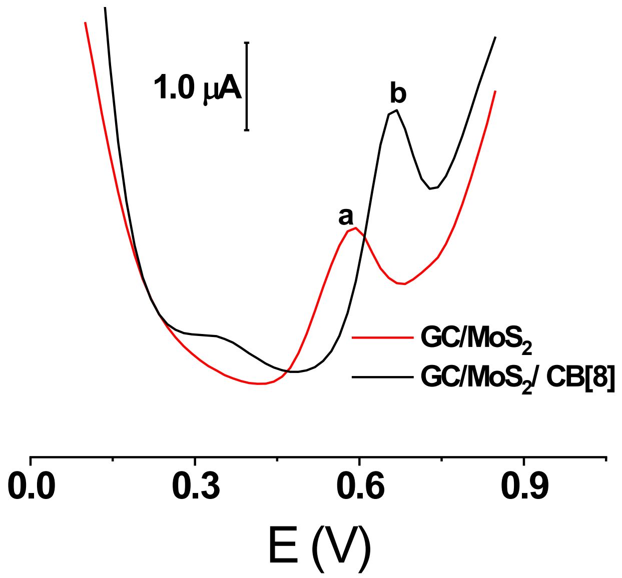

Figure S4. Differential Pulse Voltammograms of 1× 10^{-5}M MLT in phosphate buffer pH = 7 recorded with a GC/MoS_2 electrode (a) and with a GC/MoS_2/CB[8] electrode (b)

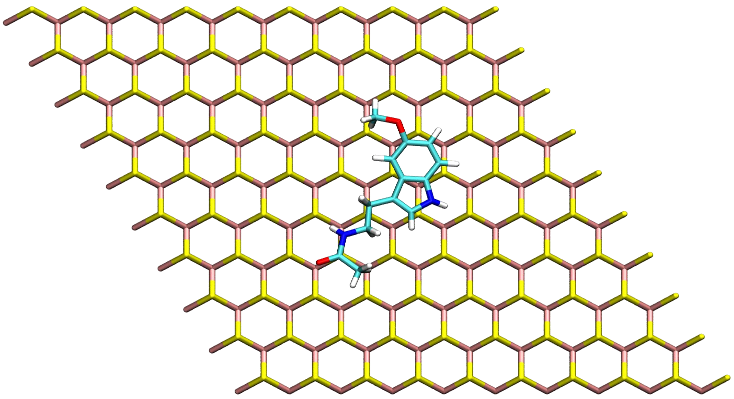

Figure S5. Structure of melatonin in a MoS_2 surface (MLT/MoS_2).

Table S1. Computed absolute energy difference of MLT.

	ΔEabs / eV
MLT/MoS_2	5.32
MLT/ MoS_2/CB[8]	5.34

4. CB[8] on graphite by AFM

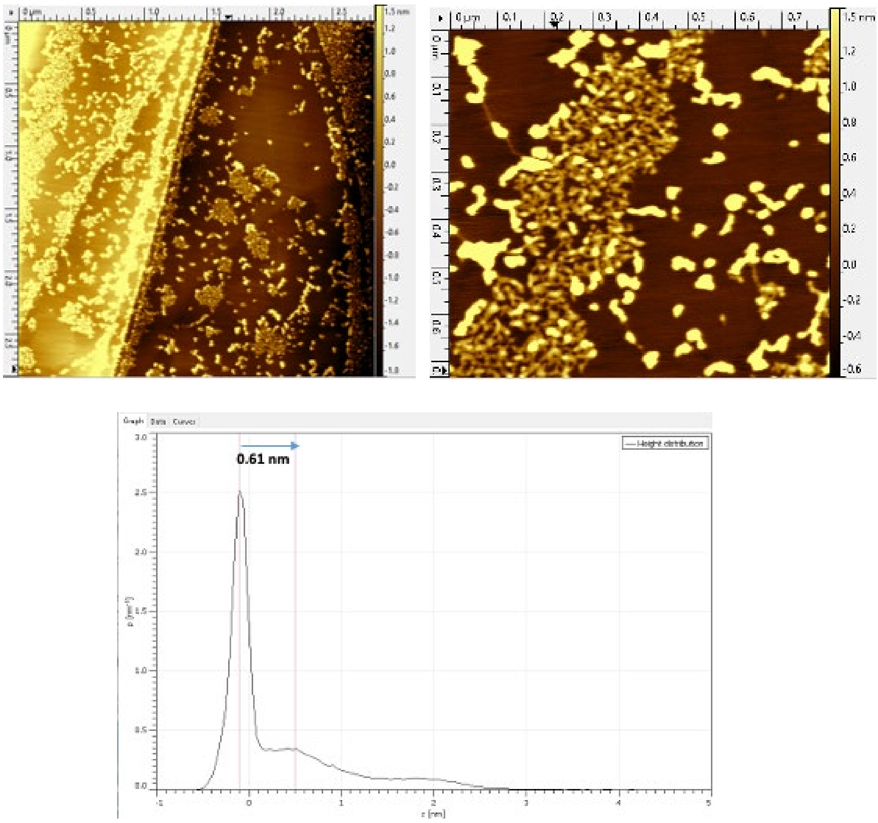

Figure S6. Structure CB[8] on graphite depicted by AFM.

The upper part of **Fig. S6** shows two AFM images of CB[8] deposited on graphite following the same protocol than in **Fig. 3**. The images have been recorded with different field of view (3 µm and 1 µm, for left and right images, respectively). These images clearly show that the molecules aggregate laterally in molecular islands that nucleate from the steps. The height of the molecular layer was estimated by a height-histogram. It shows that the distance from more intense peak, corresponding to the graphite substrate level, and the second peak, corresponding to the molecular layer, is about 0.6 nm, in good agreement with the calculations summarized in **Fig. 3B**.

References

[1] D. Nečas, P. Klapetek, *Cent. Eur. J. Phys.* **2012**, *10*, 181-188.

[2] J.P. Lewis, P. Jelínek, J. Ortega, A.A. Demkov, D.G. Trabada, B. Haycock, H. Wang, G. Adams, J.K. Tomfohr, E. Abad, H. Wang, D.A. Drabold, *Phys. Status Solidi B* **2011**, *248*, 1989-2007.

[3] C.Lee, W. Yang, R.G. Parr, *Phys. Rev. B : Condens. Matter Mater. Phys.* **1988**, *37*, 785-789.

[4] S. Grimme, S. Ehrlich, L. Goerigk, J. Comput. Chem. 2011, 32, 1456-1465.

[5] M. A. Basanta, Y. J. Dappe, P. Jelínek, J. Ortega, *Comput. Mater. Sci.* **2007**, *39*, 759–766

[6] W. L. Jorgensen, J. Chandrasekhar, J. D. Madura, R. W. Impey, M. L. Klein, *J. Chem. Phys.* **1983**, *79*, 926–935

[7] J.I. Mendieta-Moreno, R.C. Walker, J.P. Lewis, P. Gómez-Puertas, J. Mendieta, J. Ortega, J. Chem. Theory Comput. 2014, 10, 2185-2193.

[8] Marcos-Alcalde I, Mendieta-Moreno JI, Puisac B, Gil-Rodriguez MC, Hernandez-Marcos M, Soler-Polo D, Ramos FJ, Ortega J, Pie J, Mendieta J, Gomez-Puertas P (2017) Two-step ATP-driven opening of cohesin head. Sci Rep 7(1):3266

[9] B. R. Miller, T. D. McGee, J. M. Swails, N. Homeyer, H. Gohlke, A. E. Roitberg, *J. Chem. Theory Comput.* **2012**, *8*, 3314-3321.

[10] Gaussian 16, Revision C.01, M. J. Frisch, G. W. Trucks, H. B. Schlegel, G. E. Scuseria, M. A. Robb, J. R. Cheeseman, G. Scalmani, V. Barone, G. A. Petersson, H. Nakatsuji, X. Li, M. Caricato, A. V. Marenich, J. Bloino, B. G. Janesko, R. Gomperts, B. Mennucci, H. P. Hratchian, J. V. Ortiz, A. F. Izmaylov, J. L. Sonnenberg, D. Williams-Young, F. Ding, F. Lipparini, F. Egidi, J. Goings, B. Peng, A. Petrone, T. Henderson, D. Ranasinghe, V. G. Zakrzewski, J. Gao, N. Rega, G. Zheng, W. Liang, M. Hada, M. Ehara, K. Toyota, R. Fukuda, J. Hasegawa, M. Ishida, T. Nakajima, Y. Honda, O. Kitao, H. Nakai, T. Vreven, K. Throssell, J. A. Montgomery, Jr., J. E. Peralta, F. Ogliaro, M. J. Bearpark, J. J. Heyd, E. N. Brothers, K. N. Kudin, V. N. Staroverov, T. A. Keith, R. Kobayashi, J. Normand, K. Raghavachari, A. P. Rendell, J. C. Burant, S. S. Iyengar, J. Tomasi, M. Cossi, J. M. Millam, M. Klene, C. Adamo, R. Cammi, J. W. Ochterski, R. L. Martin, K. Morokuma, O. Farkas, J. B. Foresman, and D. J. Fox (Gaussian, Inc., Wallingford CT, 2016).

[11] J. Tomasi, B. Mennucci, R. Cammi, *Chem. Rev.* **2005**, *105*, 2999-3094.

[12] M. del Pozo, R. Sanchez-Sanchez, L. Vazquez, E. Blanco, M.D. Petit-Dominguez, J.A. Martin-Gago, E. Casero, C. Quintana, *Microchim. Acta* **2019**, *186*, 793.

[13] J.-H. Chen, L.-M. He and R. L. Wang, J. Phys. Chem. A **2013**, *117*, 5132–5139.

Author contributions

R.M-M and M. P. made the electrochemical experiments, L.V. recorded and analysed AFM images, S.C., and J.M. performed the calculations, A.C. performed the NMR experiments. All authors discussed the results and participated in the paper preparation and editing. The research was coordinated by J.A.M.-G., E.C., M.D.P-D. and C.Q.
